# Transcriptome Profiling Based at Different Time Points after Hatching Deepened Our Understanding on Larval Growth and Development of *Amphioctopus fangsiao*

**DOI:** 10.3390/metabo13080927

**Published:** 2023-08-08

**Authors:** Zan Li, Xiaokai Bao, Xiumei Liu, Weijun Wang, Jianmin Yang, Xibo Zhu, Shuhai Wang

**Affiliations:** 1 School of Agriculture, Ludong University, Yantai 264025, China; 2 College of Life Sciences, Yantai University, Yantai 264005, China; 3 Fishery Technology Service Center of Lanshan District, Rizhao 276800, China; 4 Ocean and Aquatic Research Center of Hekou District, Dongying 257200, China

**Keywords:** *Amphioctopus fangsiao*, larval growth and development, protein–protein interaction network, transcriptome

## Abstract

As the quality of life improves, there is an increasing demand for nutrition-rich marine organisms like fish, shellfish, and cephalopods. To address this, artificial cultivation of these organisms is being explored along with ongoing research on their growth and development. A case in point is *Amphioctopus fangsiao*, a highly valued cephalopod known for its tasty meat, nutrient richness, and rapid growth rate. Despite its significance, there is a dearth of studies on the *A. fangsiao* growth mechanism, particularly of its larvae. In this study, we collected *A. fangsiao* larvae at 0, 4, 12, and 24 h post-hatching and conducted transcriptome profiling. Our analysis identified 4467, 5099, and 4181 differentially expressed genes (DEGs) at respective intervals, compared to the 0 h sample. We further analyzed the expression trends of these DEGs, noting a predominant trend of continuous upregulation. Functional exploration of this trend entailed GO and KEGG functional enrichment along with protein–protein interaction network analyses. We identified GLDC, DUSP14, DPF2, GNAI1, and ZNF271 as core genes, based on their high upregulation rate, implicated in larval growth and development. Similarly, CLTC, MEF2A, PPP1CB, PPP1R12A, and TJP1, marked by high protein interaction numbers, were identified as hub genes and the gene expression levels identified via RNA-seq analysis were validated through qRT-PCR. By analyzing the functions of key and core genes, we found that the ability of *A. fangsiao* larvae to metabolize carbohydrates, lipids, and other energy substances during early growth may significantly improve with the growth of the larvae. At the same time, muscle related cells in *A. fangsiao* larvae may develop rapidly, promoting the growth and development of larvae. Our findings provide preliminary insights into the growth and developmental mechanism of *A. fangsiao*, setting the stage for more comprehensive understanding and broader research into cephalopod growth and development mechanisms.

## 1. Introduction

Cephalopods, globally distributed marine mollusks, predominantly inhabit the deep sea. Characterized by their sophisticated nervous systems and adept swimming abilities, they prove challenging to be captured in large amounts [1,2,3]. As consumer demand surpasses the quantity caught, research into the artificial breeding of cephalopods and their growth and development becomes increasingly critical. *Amphioctopus fangsiao*, an economically significant cephalopod, is recognized for its rapid growth, abbreviated life cycle, and high nutritional value [4,5,6]. In spite of this, research into *A. fangsiao* has chiefly focused on its survival circumstances, dietary practices, and antibacterial activity, with limited studies concentrating on its growth and development [7,8].

The larval stage is generally characterized by its relative vulnerability [9,10]. The quality of growth among larvae directly contributes to the quality of life in the adult stage, affecting factors such as vitality and physique. Existing research illustrates that the transcriptome can be effectively used to identify crucial genes and pathways during larval growth and development. For example, Bassim et al. used transcriptomic methods to identify 16 genetic regulators linked to the early growth of *Mytilus edulis* larvae [11]. Similarly, eight distinct genes associated with the early development and metamorphosis of *Sinonovacula constricta* larvae were discovered by Niu et al. [12]. Huan et al. utilized transcriptomics to investigate the mechanisms of growth and development in *Meretrix meretrix* larvae at various stages [13]. In the same vein, transcriptome analysis can aid in identifying the key genes involved in the growth and development of *A. fangsiao* larvae.

This study involves the collection of *A. fangsiao* larvae at intervals of 0 h, 4 h, 12 h, and 24 h. At each juncture, nine larvae have been randomly partitioned into three groups for the purpose of serving as triplicate biological replicates. Following RNA extraction, an analysis of the larvae’s transcriptome was conducted, which encompassed library construction, gene functional annotation, a differential expression analysis, and trend analysis, functional enrichment analysis, as well as protein–protein interaction (PPI) network analysis. Subsequently, the expression levels of 13 genes, which regulate larval growth and development, were validated using quantitative RT-PCR (qRT-PCR). These results have deepened our knowledge of the growth model of *A. fangsiao* and have offered insight into molecular mechanisms of cephalopods.

## 2. Materials and Methods

### 2.1. Sample Collection and RNA Preparation

Mature *A. fangsiao* specimens carrying eggs were identified and collected from the Rizhao Sea region. Following a short period of nurturing, these specimens produced eggs, which were safeguarded meticulously until they hatched. The eggs were incubated in flowing seawater maintained at a temperature range of 19–20.8 °C, leading to hatching after 29 days. Subsequently, the larvae were held transiently for a duration of 24 h in flowing seawater. A sample of larvae was then gathered at intervals of 0 h, 4 h, 12 h, and 24 h post-hatching, promptly frozen in liquid nitrogen, and secured for RNA extraction employing the TRIzol technique. Nine lively and robust *A. fangsiao* larvae were arbitrarily picked from each group for RNA extraction. From these, three larvae were randomly chosen at each time interval, and an equivalent molar mass of RNA was condensed in each of the replicates for constructing the transcriptome library. The same procedure was adopted to amalgamate equivalent molar masses of RNA from the trio of larvae into the second replicate. The equilibrium larval molar mass RNA was focused in the third replicate. Ultimately, the residual RNA was conserved for qRT-PCR validation.

### 2.2. Library Construction and Illumina Sequencing

The RNA-seq process was executed at Novogene Co., Ltd. (Beijing, China) as per the protocol described by Li et al. [14]. Sample libraries were constructed using the NEBNext^®^ Ultra™ RNA Library Prep Kit for Illumina^®^ (San Diego, CA, USA). The process involved several discrete steps. First, mRNA was extracted from total RNA using poly-T oligo-attached magnetic beads. The purified mRNA was then fragmented within the fragmentation buffer. In the subsequent stage, the first-strand cDNA was synthesized using random hexamers. Additionally, second-strand cDNA was generated in a buffer containing dNTPs, DNA polymerase I, and RNase H. After this, the cDNA underwent several processes such as purification, end repair, poly-A linking, and adaptor ligation. The cDNA was then amplified via PCR, and AMPure XP beads were deployed for product purification. Finally, samples across four larval group periods (0 h, 4 h, 12 h, and 24 h) were sequenced using an Illumina HiSeq 4000 platform, with the detailed data presented in Table S1.

### 2.3. De Novo Assembly

The NCBI Short Read Archive (SRA) database received the raw data submission, identified by accession numbers ranging from SRR15204591 to SRR15204602. To enhance the precision of the de novo assembly outcomes, we removed any reads that included adapters, exceeded 10% in unknown nucleotides, or had more than 50% Q-value at or below 20 bases. Clean reads, following these eliminations, underwent assembly with Trinity. Initially, Inchworm facilitated contig assembly from the clean reads. Minimal overlap contigs were then grouped into components through Chrysalis’ aid. Using these contigs, Butterfly established the transcripts, which were subsequently organized into clusters. Within each cluster, we identified the lengthy transcript as a unigene. The Trinity operation utilized a k-mer length of 25 and opted to use default parameters.

### 2.4. Gene Expression Level Analysis and Function Annotation

The functions of unigenes were determined by annotating them using NR, NT, SwissProt, Pfam, GO, and KOG public databases. They were identified through BLASTX with an E_value cut-off of ≤1 × 10^−5^ for NR, NT, SwissProt, and KOG annotations; the Hmmer 3.0 package with an E_value of ≤0.01 for Pfam annotation and GO functional annotation were conducted both by the Blast2GO program and WEGO software. Clean reads were then mapped to the assembled transcriptome, utilizing FPKM to quantify RNA-seq gene expression levels. Differentially expressed genes (DEGs) were pinpointed through the usage of DESeq2 software. Results were refined for multiple testing, employing an FDR parameter of <0.01. DEGs, denoted as genes with an absolute log_2_ fold change of ≥1 and a *p*-value of ≤0.05, were taken into account. The common points of these DEGs were used for further analysis.

### 2.5. Trend Analysis and Identification of Core Gene

The gene expression trend distribution was discerned via trend analysis. Our research entailed the utilization of the STEM method for analyzing and clustering DEGs’ trends. Within this method, the Maximum Unit Change in model profiles between time points was defined as 1, and the Maximum Output Profiles Number was set at 10, wherein identical profiles were consolidated. Further, the DEG fold changes were set as no less than 2.0, and significant trends with a threshold *p*-value of equal to or less than 0.05 were filtered [15]. Subsequently, the trend of utmost significance was filtered. This trend was used to construct fitting curves for gene expression where the five genes with the steepest slope of the fitting curve were identified as core genes.

### 2.6. Functional Enrichment Analyses

The DAVID v6.8 software was employed to enrich DEGs into the GO terms and KEGG signaling pathways. All annotated genes were considered the background gene set. Meanwhile, DEGs that showed the most significant trend were utilized as a validation set to investigate the functions of DEGs in regulating growth and development. Subsequently, the DEGs were enriched into KEGG signaling pathways and GO terms linked to the biological processes, molecular function, and cellular components. Eventually, significantly enriched GO terms and KEGG signaling pathways were identified to further understand the mechanism of *A. fangsiao* larval growth and development.

### 2.7. Functional Protein Association Networks Construction

DEGs that were significantly enriched were utilized to construct a PPI network via STRING v11.0, using default parameters. Ten DEGs characterized by high protein interaction numbers were chosen and considered core genes in controlling the growth and development of *A. fangsiao* larvae. Among them, the five DEGs with the top protein interaction numbers were identified as hub genes likely to govern larval growth and development. This methodology was outlined by Szklarczyk et al. [16]. In brief, protein sequences were initially provided to STRING and then mapped to its database. Following this, proteins were identified based on their functions, which were used in the following network construction. The final step involved adjusting parameters and removing proteins that did not interact with any other proteins.

### 2.8. Quantitative RT-PCR Validation

The expression levels of 13 genes which regulate larval growth and development were verified utilizing qRT-PCR. Gene-specific primers were created using the software Primer Premier 5.0. The DEGs and corresponding primer sequences can be observed in Table S2. The stabilities of β-actin, 18S, and GAPDH genes throughout various stages of *A. fangsiao* embryonic development and across different tissue types were assessed. Notably, β-actin served as the endogenous control because of its stable expression. The qRT-PCR methodology employed was based on the protocol described by Li et al. [17].

## 3. Results

### 3.1. Distribution and Expression Analysis of DEGs

**Figure 1:**
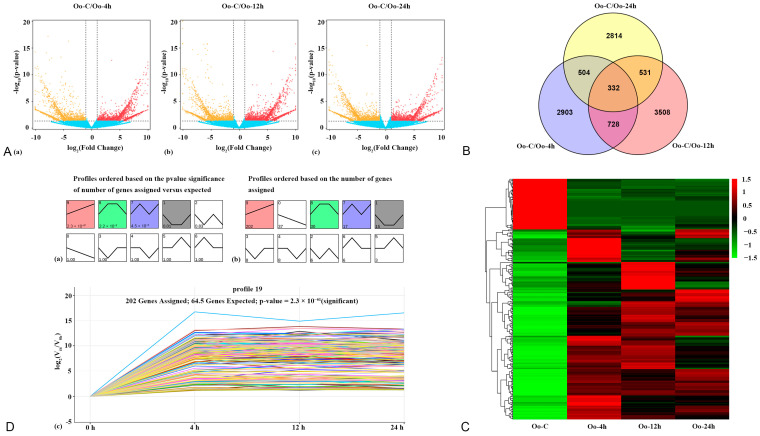
The screening, hierarchical clustering, and expression trends analyses of DEGs. (**A**), The volcano plot shows the screening and expression of DEGs. (**a**) DEG distributions between Oo-C and Oo-4h. Each dot represents a gene. The downregulated DEGs are shown as yellow dots; the upregulated DEGs are shown as red dots; and the indifferent genes are shown as blue dots. (**b**) DEG distributions between Oo-C and Oo-12h. (**c**) DEG distributions between Oo-C and Oo-24h. (**B**), The Venn diagram shows DEG distributions between groups. Blue represents DEGs identified only at 4 h (2903); red indicates DEGs identified only at 12 h (3508); yellow stands for DEGs identified only at 24 h (2814). DEGs identified at 4 and 12 h are displayed in purple (728); DEGs identified at 4 and 24 h are displayed in green (504); DEGs identified at 12 and 24 h are displayed in orange (531). Dark green represents that DEGs are differentially expressed at all three time points (332). (**C**), The DEG hierarchical clustering heatmap at each time point. Each row represents a gene, and each column represents a group. Colors represent DEG expressions. Red represents upregulation, and green stands for down-regulation. (**D**), Analysis of DEGs expression trends. (**a**) Four of the 10 trends are significantly enriched (*p*-value ≤ 0.05) and represented by different colors. A trend without color means it is not significantly enriched. (**b**) DEG numbers enriched in each trend. (**c**) Expression trend of DEGs in Profile 9. 202 DEGs are enriched in this trend, and the *p*-value is 2.3 × 10^−61^. The *x*-axis stands for larval growth time after hatching and the *y*-axis represents log_2_ (fold change).

The results of the differential expression analysis revealed that there were 4467, 5099, and 4181 differentially expressed genes (DEGs) identified at 4 h, 12 h, and 24 h post-hatching, respectively, in comparison with the baseline at 0 h. Out of these DEGs, we observed 2270 upregulated and 2197 down-regulated DEGs at 4 h; 2729 upregulated and 2370 down-regulated DEGs at 12 h; and 1637 upregulated and 2544 down-regulated DEGs at 24 h (Figure 1A). The Venn diagram displayed 332 DEGs that were differentially expressed at all three time points, which were used for subsequent analysis (Figure 1B). The heatmap helped visualize the clustering distribution of DEGs (Figure 1C). The expression levels of DEGs at different time points had significant variations, and a considerable quantity of DEGs were upregulated compared to the larvae at 0 h.

### 3.2. Trend Analysis of DEGs

We analyzed the expression trends of DEGs in Figure 1B using the STEM method. Out of the ten trends, four showed significant expression trends (*p*-value < 0.05) as represented in Figure 1D. The most noteworthy trend was Profile 9 (presented in Figure 1(Dc)) having the smallest *p*-value (2.3 × 10^−61^), suggesting its high significance. This profile contained the maximum number of DEGs (202) and was deemed the most pertinent to the growth and development of *A. fangsiao* larvae. Of these DEGs, 13 genes showed a consistent upregulation alongside the growth of *A. fangsiao* larvae. The expression levels of these genes were utilized to construct the fitting curves (refer to Table S3). Out of the five genes—GLDC, DUSP14, DPF2, GNAI1, and ZNF271—with the steepest curve slope, they were identified as the key genes responsible for larval growth and development.

### 3.3. GO and KEGG Functional Enrichment Analyses

**Figure 2:**
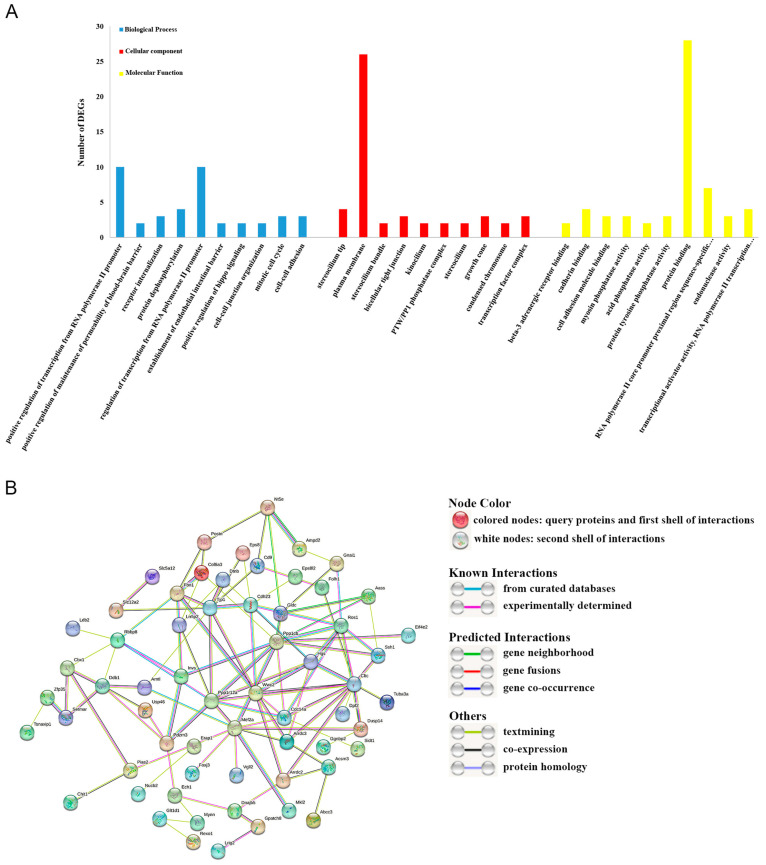
Results of GO functional enrichment and PPI network analyses. (**A**), GO classifications of DEGs in *A. fangsiao* larvae within 24 h of growth and development. The *y*-axis represents DEG numbers involved in 10 significantly enriched GO terms of each category; the *x*-axis indicates specific GO terms. (**B**), PPI network based on 202 DEGs enriched in Profile 9. Each node represents a protein. Different edges represent different relationships between proteins, which are described in the legends below the network.

The top 10 level-3 terms within the three designated categories namely, Biological Process (BP), Molecular Function (MF), and Cellular Component (CC), were identified through GO enrichment analysis as depicted in Figure 2A. These terms comprise cell–cell adhesion, cell–cell junction organization, positive activation of Hippo signaling, and mitotic cell cycle, among others, all of which have significant relevance to growth and development. Concurrently, two notable KEGG signaling pathways, the endocytosis signaling pathway and metabolic pathways, were also identified.

### 3.4. Construction of the PPI Network

We utilized 202 DEGs’ protein sequences to construct PPI networks. Our intent was to identify key genes closely associated with larval growth and development, as illustrated in Figure 2B. Through our method, we found ten key genes that exhibited a high number of protein interactions, as shown in Table 1. Out of these, five genes—CLTC, MEF2A, PPP1CB, PPP1R12A, and TJP1—displayed the highest number of protein interactions and were hence identified as hub genes. Specific parameters of the network are provided in Supplementary Table S4.

### 3.5. qRT-PCR Verification

**Figure 3:**
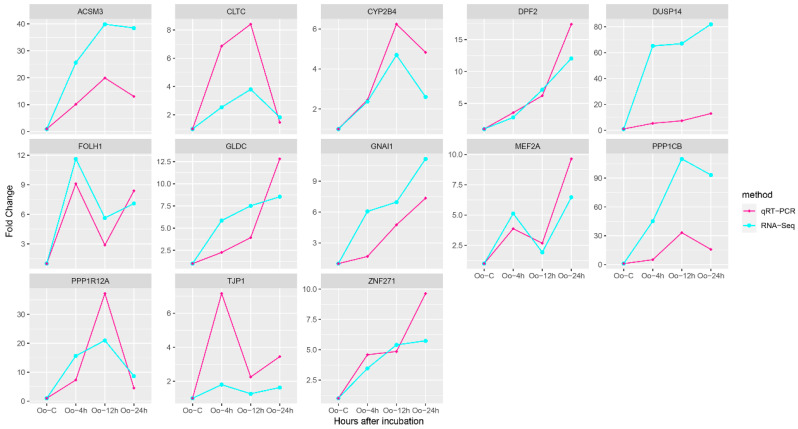
The relative expression level verification of hub and core genes regulating larval growth and development using qRT-PCR. We normalized Ct values of these 13 genes to the *β-actin* Ct value to determine their relative fold differences. Gene expression levels of Oo-C are normalized to 1, and relative expression levels are calculated relative to a calibrator using a formula 2^−ΔΔCt^. The *x*-axis indicates times of larval growth after hatching; the *y*-axis represents fold changes of gene expressions at four time points.

We executed a quantitative evaluation of the relative expression levels for 13 genes, which ostensibly regulate larval growth and development, using qRT-PCR. Our data revealed that all DEGs measures yielded single products. Remarkably, the results from qRT-PCR and RNA-Seq displayed significant correlation. Moreover, the gene expression trends acquired via these two methodologies were found to be parallel, as depicted in Figure 3.

## 4. Discussion

### 4.1. DEG Expression Trend Analysis

Compared to the entire collection of differentially expressed genes (DEGs), the individual genes with differential expression at distinct time points are more likely to play pivotal roles in the growth and development of *A. fangsiao* larvae. Trend analysis indicates a continuous upregulation in the expression of most genes; the interplay between these genes possibly facilitates the insects’ development. Five critical genes—GLDC, DUSP14, DPF2, GNAI1, and ZNF271—showed continuous upregulation effectively regulate larval growth and development.

GLDC, a key enzyme in the glycine cleavage system, is involved in glycine metabolism and the conversion of glycine into a one-carbon unit, thereby enhancing glucose metabolism, energy metabolism, and other metabolic processes [18,19,20]. Prior research demonstrates that elevated expression of GLDC boosts cell proliferation, reinforces cell antioxidant capacity, improves cell survival, and significantly expedites the development of an organism’s tissues and organs [21,22,23]. Consequently, the uninterrupted upregulation of GLDC likely enhances the growth and development of *A. fangsiao* larvae by boosting cell proliferation and metabolic processes, like glycine metabolism, glucose metabolism, and energy metabolism.

Dual-specificity phosphatases (DUSPs) are enzymes that dictate metabolism and cell-based processes such as growth and differentiation [24]. DUSP14, a crucial DUSP predominantly found in the liver, invokes glucose and lipid metabolism by regulating the MAPK signaling pathway, thereby maintaining liver metabolic equilibrium [25,26,27]. It also facilitates the proliferation and differentiation of liver cells, diminishes cell mortality, and stimulates liver development [28]. In this study, DUSP14 was continuously upregulated with the growth of larvae, indicating that DUSP14 may be a core gene that regulates liver energy metabolism and promotes liver cell growth and development.

The role of gene expression regulation in organismal growth is fundamental. Both DPF2 and ZNF271 are zinc-finger domain carrying proteins. Existing research indicates that DPF2 functions as a transcription factor regulating cell apoptosis and DNA-protein binding [29]. ZNF271, however, lacks elucidated functionality. These two genes are significantly involved in the positive regulation of transcription from the RNA polymerase II promoter term and the RNA polymerase II promoter term regulation. Unsystematic gene expression can trigger a cascade of pathological reactions, so regularity in expression is vital [30]. The transcription of RNA polymerase II promoter is crucial for gene expression and regulation in organisms. It mediates cell growth, development, and differentiation by controlling the timing and level of gene expression [31,32,33]. In addition, it governs the cell cycle and growth factor expression, thereby promoting growth and development in organisms [34,35]. As DPF2 and ZNF271 exhibit continuous upregulation, we hypothesize that they likely influence the cellular process and gene expression to foster *A. fangsiao* larvae growth and development.

GNAI1, a chief protein regulating cell signal transduction, significantly impacts the regulation of cellular processes like proliferation, migration, and differentiation [36,37]. It is ubiquitously expressed in organisms, sustains the stability of the nervous and motor systems, and facilitates growth [38,39]. The continuous upregulation of GNAI1 may favor proliferation, differentiation, signal transduction, and other cellular processes, as well as enhance the nervous system, and boost the physical agility of the *A. fangsiao* larvae.

Conclusively, the continuously upregulated genes potentially promote *A. fangsiao* larvae development through the regulation of cell growth, differentiation, survival, activation of growth factors, and the augmentation of glucose and lipid metabolism. The precise roles that these genes play in *A. fangsiao* larvae, however, remain ill-defined and warrant further investigation in subsequent experiments.

### 4.2. Enrichment Analysis of GO Terms KEGG Signaling Pathways

Our functional enrichment analysis, based on GO and KEGG, has identified several terms and signaling pathways relevant to growth and development. A case in point is the upregulated terms of cell–cell adhesion and cell–cell junction organization. These findings suggest an enhanced cell–cell and cell-matrix adhesion, promoting cellular proliferation, differentiation, migration, signal transduction, and transmembrane transport, thus fostering tissue development in larval stages of *A. fangsiao* [40,41,42,43,44]. Similarly, the observed enrichment of the positive regulation of Hippo signaling strengthens the notion of promoted cellular proliferation and differentiation, gradually stabilizing tissue function and accelerating growth and development in *A. fangsiao* larvae [45,46,47,48]. Further evidence of rapid growth is provided by the identification of the mitotic cell cycle term, indicating enhanced cell proliferation and organ development in *A. fangsiao* larvae [49,50]. The above results indicate that the growth of *A. fangsiao* larvae during early development may be regulated by a complex network, and cellular processes such as proliferation and differentiation may be the core processes of network regulation.

Endocytosis, a universal cellular process evident across varying tissues, governs transmembrane transport and cellular signal transduction. This regulation, impacting the binding and internalization of extracellular macromolecules and cell surface receptors, influences cell proliferation, polarization, and migration [51]. Endocytosis also mediates the synthesis of proteins, DNA, and lipids, thereby facilitating cell growth [52]. Recent scholarship suggests a strong connection between endocytosis and the activation and regulation of growth factors. These growth factors heighten early larval development and preserve tissue stability by manipulating cell proliferation, differentiation, survival, and migration [53,54]. Endocytosis, in its capacity as a signal regulator, modulates the transference of growth factor receptor signals to control the expression of growth factors, thus fostering organismal growth and development [55]. In this study, the upregulation of the endocytosis signaling pathway suggests heightened endocytosis activity in *A. fangsiao* larvae. This may result in large-scale expression of growth factors, potentially significantly enhancing larval growth and development whilst retaining tissue stability.

Metabolic stability plays a crucial role in growth and development. The notable enrichment of metabolic pathways in *A. fangsiao* larvae suggests an accelerated metabolic process, indicating rapid growth. Specifically, three genes—ACSM3, CYP2B4, and FOLH1—are enriched within this pathway. ACSM3 is primarily involved in two metabolic processes: regulating fatty acid metabolism as an early stage key enzyme [56,57], and participating in butyric acid metabolism. This gene converts butyric acid into butyryl-CoA for regulating polyketone biosynthesis and expression of growth factors, thus contributing to the growth of organisms [58]. CYP2B4, an endoplasmic reticulum resident protein, has a significant role in drug metabolism [59] and also ensures metabolic stability by catalyzing the metabolism of various substrates and optimizing some protein functions [60,61]. FOLH1 participates in the metabolism of folic acid compounds, maintaining the stability of glucose and lipid metabolism [62,63]. Thus, it can be inferred that the metabolic processes involving fatty acids, butyric acids, folic acids, and other processes in early stage *A. fangsiao* larvae are enhanced, likely promoting developmental growth through the upregulation of growth factors.

### 4.3. Speculation of Hub Genes

Proteins synergistically regulate the growth and development of organisms. Central proteins in the interaction network often play crucial roles in biological processes. This study identifies five genes—CLTC, MEF2A, PPP1CB, PPP1R12A, and TJP1—each one exhibiting a high number of protein interactions, potentially functioning as hub genes for larval growth and development regulation.

CLTC, a clathrin coated scaffold protein, has a pivotal role in endocytosis, promoting intercellular material transport and signal transduction by internalizing extracellular substances on the plasma membrane [60,64,65]. CLTC also regulates muscle tissue growth and development. An elevated expression of CLTC augments the proliferation and differentiation of muscle cells, increases muscle tension, and improves swimming power [65,66,67]. MEF2A, akin to CLTC, participates in musculature development regulation. It directs the expression of growth factors and triggers both WNT and MAPK signaling pathways, governing numerous cellular processes in muscle cells such as proliferation, differentiation, growth, migration, and apoptosis. This, in turn, bolsters the muscle tissue growth and development [68,69,70,71]. The substantial upregulation of CLTC and MEF2A demonstrated in this study insinuates that these processes may enhance the motility of *A. fangsiao* larvae.

Protein phosphatase type 1 (PPP1), a pivotal phosphatase ubiquitously present across many tissues, regulates cell growth, differentiation, and muscle development along with intercellular signal transduction [72,73,74]. It consists of catalytic subunit PPP1C, and regulatory subunit PPP1R [75]. Specifically, PPP1CB, predominantly expressed in muscle, promotes muscle tissue growth through its involvement in muscle cell proliferation and differentiation [76,77]. PPP1R12A regulates muscle cell characteristics such as growth, cycle, adhesion, and migration, and has a pivotal role in the control of muscle contraction and relaxation [78,79]. These results propose that PPP1CB and PPP1R12A have instrumental roles during early locomotor system development in *A. fangsiao* larvae, regulating muscle growth and development from multiple perspectives.

TJP1, a membrane-associated cell barrier protein, influences growth factor expression and signal transduction between cells by controlling cell connection and adhesion [80]. Further, it participates in managing the protein network’s structure between actin as well as the tight junction protein overall, thus maintaining cell structure and function stability [81]. For instance, in epithelial cells TJP1 joins transmembrane proteins and the cytoskeleton to form scaffold proteins, facilitating epithelial cell growth and modulating cell proliferation and differentiation [82,83,84]. A notable upregulation of TJP1 suggests the cell–cell interaction significantly tightens during the early growth stage in *A. fangsiao* larvae, facilitating rapid proliferation and differentiation while upholding cell integrity, thus fostering tissue development.

In conclusion, we discovered that the central genes in the PPI network are significantly correlated with muscle tissue growth and development. The primary reason might be the initially poor mobility of hatched *A. fangsiao* larvae. As the muscle development progresses, this effectively increases the swimming and predatory capabilities of the larvae, thereby enhancing environmental adaptability.

## 5. Conclusions

We analyzed the growth mechanism of *A. fangsiao* larvae within 24 h post hatching using transcriptome analysis, pinpointing ten principle genes: GLDC, DUSP14, DPF2, GNAI1, ZNF271, CLTC, MEF2A, PPP1CB, PPP1R12A, and TJP1. Our findings suggest that fundamental cellular processes in *A. fangsiao* larvae, such as growth, proliferation, differentiation, as well as metabolic processes involving glucose, folic acid, glycine, are enhanced. Of noteworthy mention is the rapid development of larval muscle tissue, potentially augmenting their swimming and predation capabilities. These results furnish insight into the growth mechanisms of octopus larvae, underscoring a solid foundation for future exploration of cephalopod physiological processes. The muscle development and metabolic rate of *A. fangsiao* larvae are very fast, which may require vast swimming space and suitable bait. We suggest feeding bait that is beneficial for muscle growth and contains rich metabolic materials during the artificial breeding process of *A. fangsiao* larvae, as well as providing reasonable breeding space. These measures will be conducive to the rapid growth of octopus larvae.

## Figures and Tables

**Table 1:** Summary of key and hub DEGs.

Gene Name (Abbreviation)	Gene Name (Official Full Name)	Number of Protein–Protein Interactions
*CLTC*	clathrin heavy chain	12
*MEF2A*	myocyte enhancer factor 2A	10
*PPP1CB*	protein phosphatase 1 catalytic subunit beta	10
*PPP1R12A*	protein phosphatase 1 regulatory subunit 12A	9
*TJP1*	tight junction protein 1	9
*ROS1*	ROS proto-oncogene 1, receptor tyrosine kinase	8
*WWC2*	WW and C2 domain containing 2	8
*CDC14A*	cell division cycle 14A	7
*CDH23*	cadherin related 23	7
*DDB1*	damage specific DNA binding protein 1	6

Number of protein–protein interactions: the interaction numbers between a protein corresponding to the gene and other proteins in the network.

## Data Availability

The original contributions presented in the study are publicly available in NCBI using accession numbers SRR15204591–SRR15204602 at the following link: https://www.ncbi.nlm.nih.gov/Traces/study/?acc=PRJNA748210&o=acc_s%3Ad (accessed on 30 May 2023).

## Supplementary Materials

The following supporting information can be downloaded at: https://www.mdpi.com/article/10.3390/metabo13080927/s1, Table S1: Summary of sequencing results; Table S2: Primer list for quantitative RT-PCR verification; Table S3: Continuous upregulation of DEG expression; Table S4: Network statistics of PPI network.
