# Supplementary material for: Transcriptome Profiling Based at Different Time Points after Hatching Deepened Our Understanding on Larval Growth and Development of Amphioctopus fangsiao
Source: Metabolites. 2023 Aug 8;13(8):927. doi: 10.3390/metabo13080927 (PMC10456336; doi:10.3390/metabo13080927)
Supplement: Supplementary file 1 [file metabolites-13-00927-s001.zip › Table S1.pdf]

Table S1. Summary of sequencing results.

Sample	Raw reads	Clean reads	Clean reads Q30 (%)	Clean reads Q20 (%)
Oo-C-1	50,581,378	49,165,388	97.54	93.27
Oo-C-2	49,281,546	46,846,720	97.42	93.07
Oo-C-3	55,727,122	53,007,342	97.39	92.94
Oo-4h-1	43,683,716	40,502,546	97.38	92.93
Oo-4h-2	50,981,374	47,814,924	97.47	93.15
Oo-4h-3	44,781,414	41,833,784	97.30	92.78
Oo-12h-1	42,605,216	40,522,842	97.72	93.70
Oo-12h-2	54,564,714	51,170,578	97.30	92.72
Oo-12h-3	56,997,500	53,933,392	97.61	93.42
Oo-24h-1	51,418,618	49,235,968	97.71	93.67
Oo-24h-2	60,787,322	58,211,806	97.52	93.20
Oo-24h-3	63,795,232	61,219,086	97.57	93.32
