# Supplementary material for: Transcriptome Profiling Based at Different Time Points after Hatching Deepened Our Understanding on Larval Growth and Development of Amphioctopus fangsiao
Source: Metabolites. 2023 Aug 8;13(8):927. doi: 10.3390/metabo13080927 (PMC10456336; doi:10.3390/metabo13080927)
Supplement: Supplementary file 1 [file metabolites-13-00927-s001.zip › Table S2.pdf]

Table S2. Primer list for quantitative RT-PCR verification.

Gene name	Forward primer (5’-3’)	TM(°C)	Reverse primer (5’-3’)	TM(°C)	Amplicon length (bp)
ACSM3	CTACTGCTCTACGCTTGTTT	60	GGCTAATCAGCTCCTCATTC	60	108
CLTC	ACATGATAGCCGAGACAGA	60	GGGTGTGATGGGTTCATTAG	60	117
CYP2B4	CCTGACGAAACACCAATGA	60	AACACACATCGACGACATAC	60	111
DPF2	CTGTGCATCGGACCATTT	60	GGTGAACTCCAGACAAAGAC	60	103
DUSP14	GGAAGCCAGAACGAGTAAAG	60	TTCACGCCATCGCTAAAG	60	133
FOLH1	CACAGAGAGAGAGAGAGAGAG	60	ACAAGGAATGATGGCTACAG	60	101
GLDC	ATCTCCTTCCAACCAAACAG	60	CCGAGATAGGGATGAGACATA	60	118
GNAI1	GGTAACAAGAGGCGGATTT	60	CTGTTCTGGGTGTGTGTATT	60	124
MEF2A	GTGCCTTCCACATCAGTATC	60	TGGCTTGGGATGGTATCT	60	106
PPP1CB	TCAGGGTCTGACCAAAGT	60	GGAGGTCTCTCTCCAGATTTA	60	110
PPP1R12A	GCCTTCAACCCTGAACTATG	60	GCAGTTGAAGCCTCTCTATG	60	101
TJP1	GCCGGAATCTAAACGTACTC	60	ATGGGTCTCCCACGTAAT	60	115
ZNF271	CACCTGTGTGAGTACGTTTATG	61	AAGAGGAGTAGAAAGCAGAGG	61	100
