# Supplementary material for: Transcriptome Profiling Based at Different Time Points after Hatching Deepened Our Understanding on Larval Growth and Development of Amphioctopus fangsiao
Source: Metabolites. 2023 Aug 8;13(8):927. doi: 10.3390/metabo13080927 (PMC10456336; doi:10.3390/metabo13080927)
Supplement: Supplementary file 1 [file metabolites-13-00927-s001.zip › Table S3.pdf]

Table S3. Continuous up-regulation of DEG expression.

Gene name (abbreviation)	Gene name (official full name)	0h expression level	4h expression level	12h expression level	24h expression level	Fitting exponential slope
GLDC	glycine decarboxylase	3.5833	20.9700	26.9433	30.5867	0.9537
DUSP14	dual specificity phosphatase 14	0.0067	6.5133	6.7000	8.2000	0.2650
DPF2	double PHD fingers 2	0.0000	0.2800	0.7133	1.2067	0.0495
GNAI1	G protein subunit alpha i1	0.0000	0.6067	0.6967	1.1167	0.0398
ZNF271	zinc finger protein 271	0.0000	0.3467	0.5400	0.5733	0.0209
ERAP1	endoplasmic reticulum aminopeptidase 1	0.0000	0.2100	0.3100	0.5300	0.0202
CDH23	cadherin related 23	0.0667	0.4433	0.5667	0.6200	0.0193
FOXJ3	forkhead box J3	0.0000	0.2333	0.3733	0.4433	0.0165
TSNAXIP1	translin associated factor X interacting protein 1	0.0000	0.2467	0.2667	0.4367	0.0154
TASOR2	transcription activation suppressor family member 2	0.0000	0.3167	0.3400	0.3933	0.0128
PIAS2	protein inhibitor of activated STAT 2	0.0000	0.0900	0.1000	0.1967	0.0072
INVS	inversin	0.0033	0.1200	0.1500	0.2033	0.0071
FBN1	fibrillin 1	0.0000	0.1733	0.1733	0.1933	0.0060
