# Supplementary material for: Transcriptome Profiling Based at Different Time Points after Hatching Deepened Our Understanding on Larval Growth and Development of Amphioctopus fangsiao
Source: Metabolites. 2023 Aug 8;13(8):927. doi: 10.3390/metabo13080927 (PMC10456336; doi:10.3390/metabo13080927)
Supplement: Supplementary file 1 [file metabolites-13-00927-s001.zip › Table S4.pdf]

Table S4. Network statistics of PPI network.

Network statistics	
Number of nodes	75
Number of edges	110
Average node degree	2.930
Clustering coefficient	0.352
Expected number of edges	101

Number of nodes: the numbers of interacting proteins.

Number of edges: the numbers of connections between proteins in the network.

Average node degree: mean value of protein interaction numbers at all nodes.

Clustering coefficient: all nodes average clustering coefficient. A single node clustering coefficient is the ratio of the actual connection numbers of its neighbors to the theoretical maximum connection numbers.

Expected number of edges: expected value of the total number of interactions between proteins in the network.
